# Supplementary material for: R-spondins engage heparan sulfate proteoglycans to potentiate WNT signaling
Source: eLife. 2020 May 20;9:e54469. doi: 10.7554/eLife.54469 (PMC7239654; doi:10.7554/eLife.54469)
Supplement: Supplementary file 1. — The name of the encoded protein and the length (in bp) of the nucleotide sequence is indicated. RSPO3 (WT), RSPO3 TSP/BR (K/R→E) and RSPO3 ΔTSP/BR were cloned into pHLsec-HA-Tev-Fc-Avi-1D4. RSPO3 ΔTSP/BR HS20, RSPO3 ΔTSP/BR HS20 (GS), RSPO3 ΔTSP/BR HS20 (A), RSPO3 ΔTSP/BR HS20 (R67A/Q72A) and RSPO3 ΔTSP/BR HS20 (F106E/F110E) were cloned into pHLsec-HA-Avi-1D4. Bases in lowercase overlap the sequences upstream of the unique AgeI sites and downstream of the unique KpnI sites in the pHLsec-HA-Tev-Fc-Avi-1D4 and pHLsec-HA-Avi-1D4 vectors, respectively. Bases in uppercase encode RSPO3 WT, mutant and HS20-fusion proteins. For mutant constructs, mutated bases are indicated in red and the resulting altered codons are underlined. For HS20-fusion constructs, bases encoding a codon-optimized glycine/serine linker (STGGSGGSGGSG) are indicated in light blue. [file elife-54469-supp1.docx]

RSPO3 (WT): 815bp

cctacgacgtgcccgactacgccaccggtaacctgCAAAACGCCTCCCGGGGAAGGCGCCAGCGAAGAATGCATCCTAACGTTAGTCAAGGCTGCCAAGGAGGCTGTGCAACATGCTCAGATTACAATGGATGTTTGTCATGTAAGCCCAGACTATTTTTTGCTCTGGAAAGAATTGGCATGAAGCAGATTGGAGTATGTCTCTCTTCATGTCCAAGTGGATATTATGGAACTCGATATCCAGATATAAATAAGTGTACAAAATGCAAAGCTGACTGTGATACCTGTTTCAACAAAAATTTCTGCACAAAATGTAAAAGTGGATTTTACTTACACCTTGGAAAGTGCCTTGACAATTGCCCAGAAGGGTTGGAAGCCAACAACCATACTATGGAGTGTGTCAGTATTGTGCACTGTGAGGTCAGTGAATGGAATCCTTGGAGTCCATGCACGAAGAAGGGAAAAACATGTGGCTTCAAAAGAGGGACTGAAACACGGGTCCGAGAAATAATACAGCATCCTTCAGCAAAGGGTAACCTGTGTCCCCCAACAAATGAGACAAGAAAGTGTACAGTGCAAAGGAAGAAGTGTCAGAAGGGAGAACGAGGAAAAAAAGGAAGGGAGAGGAAAAGAAAAAAACCTAATAAAGGAGAAAGTAAAGAAGCAATACCTGACAGCAAAAGTCTGGAATCCAGCAAAGAAATCCCAGAGCAACGAGAAAACAAACAGCAGCAGAAGAAGCGAAAAGTCCAAGATAAACAGAAATCGGTATCAGTCAGCACTGTACACttggtaccaaccaccgagaacctgtac

RSPO3 TSP/BR (K/R→E): 815bp

cctacgacgtgcccgactacgccaccggtaacctgCAAAACGCCTCCCGGGGAAGGCGCCAGCGAAGAATGCATCCTAACGTTAGTCAAGGCTGCCAAGGAGGCTGTGCAACATGCTCAGATTACAATGGATGTTTGTCATGTAAGCCCAGACTATTTTTTGCTCTGGAAAGAATTGGCATGAAGCAGATTGGAGTATGTCTCTCTTCATGTCCAAGTGGATATTATGGAACTCGATATCCAGATATAAATAAGTGTACAAAATGCAAAGCTGACTGTGATACCTGTTTCAACAAAAATTTCTGCACAAAATGTAAAAGTGGATTTTACTTACACCTTGGAAAGTGCCTTGACAATTGCCCAGAAGGGTTGGAAGCCAACAACCATACTATGGAGTGTGTCAGTATTGTGCACTGTGAGGTCAGTGAATGGAATCCTTGGAGTCCATGCACGAAGAAGGGAAAAACATGTGGCTTCGAAAGAGGGACTGAAACACGGGTCCGAGAAATAATACAGCATCCTTCAGCAAAGGGTAACCTGTGTCCCCCAACAAATGAGACAAGAGAGTGTACAGTGCAAGAGAAGGAGTGTCAGGAGGGAGAACGAGGAGAAAAAGGAGAGGAGGAGAAAGAGAAAGAACCTAATAAAGGAGAAAGTAAAGAAGCAATACCTGACAGCAAAAGTCTGGAATCCAGCAAAGAAATCCCAGAGCAACGAGAAAACAAACAGCAGCAGGAGAAGGAAAAAGTCCAAGATAAACAGAAATCGGTATCAGTCAGCACTGTACACttggtaccaaccaccgagaacctgtac

RSPO3 ΔTSP/BR: 437bp

cctacgacgtgcccgactacgccaccggtaacctgCAAAACGCCTCCCGGGGAAGGCGCCAGCGAAGAATGCATCCTAACGTTAGTCAAGGCTGCCAAGGAGGCTGTGCAACATGCTCAGATTACAATGGATGTTTGTCATGTAAGCCCAGACTATTTTTTGCTCTGGAAAGAATTGGCATGAAGCAGATTGGAGTATGTCTCTCTTCATGTCCAAGTGGATATTATGGAACTCGATATCCAGATATAAATAAGTGTACAAAATGCAAAGCTGACTGTGATACCTGTTTCAACAAAAATTTCTGCACAAAATGTAAAAGTGGATTTTACTTACACCTTGGAAAGTGCCTTGACAATTGCCCAGAAGGGTTGGAAGCCAACAACCATACTATGGAGTGTGTCAGTATTGTGttggtaccaaccaccgagaacctgtac

RSPO3 ΔTSP/BR HS20: 1188bp

cctacgacgtgcccgactacgccaccggtaacctgCAAAACGCCTCCCGGGGAAGGCGCCAGCGAAGAATGCATCCTAACGTTAGTCAAGGCTGCCAAGGAGGCTGTGCAACATGCTCAGATTACAATGGATGTTTGTCATGTAAGCCCAGACTATTTTTTGCTCTGGAAAGAATTGGCATGAAGCAGATTGGAGTATGTCTCTCTTCATGTCCAAGTGGATATTATGGAACTCGATATCCAGATATAAATAAGTGTACAAAATGCAAAGCTGACTGTGATACCTGTTTCAACAAAAATTTCTGCACAAAATGTAAAAGTGGATTTTACTTACACCTTGGAAAGTGCCTTGACAATTGCCCAGAAGGGTTGGAAGCCAACAACCATACTATGGAGTGTGTCAGTATTGTGAGTACCGGAGGTTCAGGGGGAAGCGGAGGTTCCGGTATGGAGGTCCAACTTCTCGAAAGCGGTGGTGGTCTTGTCCAACCAGGAGGGTCACTCCGCTTGTCCTGCGCAGCTTCTGGTTTCACTTTTTCAAGCTATGCGATGAGCTGGGTGCGCCAGGCCCCTGGGAAAGGGTTGGAATGGGTCAGCACGATACAAAAACAAGGGCTTCCAACTCAATATGCAGACAGCGTGAAAGGACGGTTTACAATTTCACGAGACAATAGCAAAAATACGTTGTATTTGCAAATGAATTCACTGCGAGCGGAAGACACAGCAGTCTATTATTGTGCAAAGAATAGGGCCAAGTTTGATTACTGGGGTCAAGGGACGCTCGTTACCGTTAGCAGTGGTGGAGGAGGGTCTGGGGGCGGCGGTAGCGGGGGAGGCGGAAGTGACATCCAGATGACACAAAGTCCTTCATCCCTGAGCGCAAGTGTTGGGGATCGCGTTACCATTACCTGCCGCGCTTCCCAAAGTATCTCTTCTTATCTCAACTGGTATCAACAAAAACCGGGCAAAGCGCCTAAGTTGTTGATCTACGCAGCGTCAATGCTCCAAAGCGGTGTTCCAAGCAGATTCTCCGGCAGTGGCTCAGGGACGGACTTTACGCTTACGATATCTAGCCTTCAGCCGGAAGACTTTGCAACATATTACTGCCAGCAGAATCGAGGCTTTCCACTTACTTTTGGGCAGGGTACGAAAGTGGAAATAAAAggtaccggaggttccggtggttccg

RSPO3 ΔTSP/BR HS20 (GS): 1188bp

cctacgacgtgcccgactacgccaccggtaacctgCAAAACGCCTCCCGGGGAAGGCGCCAGCGAAGAATGCATCCTAACGTTAGTCAAGGCTGCCAAGGAGGCTGTGCAACATGCTCAGATTACAATGGATGTTTGTCATGTAAGCCCAGACTATTTTTTGCTCTGGAAAGAATTGGCATGAAGCAGATTGGAGTATGTCTCTCTTCATGTCCAAGTGGATATTATGGAACTCGATATCCAGATATAAATAAGTGTACAAAATGCAAAGCTGACTGTGATACCTGTTTCAACAAAAATTTCTGCACAAAATGTAAAAGTGGATTTTACTTACACCTTGGAAAGTGCCTTGACAATTGCCCAGAAGGGTTGGAAGCCAACAACCATACTATGGAGTGTGTCAGTATTGTGAGTACCGGAGGTTCAGGGGGAAGCGGAGGTTCCGGTATGGAGGTCCAACTTCTCGAAAGCGGTGGTGGTCTTGTCCAACCAGGAGGGTCACTCCGCTTGTCCTGCGCAGCTTCTGGTTTCACTTTTTCAAGCTATGCGATGAGCTGGGTGCGCCAGGCCCCTGGGAAAGGGTTGGAATGGGTCAGCACGATACAAAAACAAGGGCTTCCAACTCAATATGCAGACAGCGTGAAAGGACGGTTTACAATTTCACGAGACAATAGCAAAAATACGTTGTATTTGCAAATGAATTCACTGCGAGCGGAAGACACAGCAGTCTATTATTGTAGCGGTGGAGGAAGTGGAGGAGGATCTTGGGGTCAAGGGACGCTCGTTACCGTTAGCAGTGGTGGAGGAGGGTCTGGGGGCGGCGGTAGCGGGGGAGGCGGAAGTGACATCCAGATGACACAAAGTCCTTCATCCCTGAGCGCAAGTGTTGGGGATCGCGTTACCATTACCTGCCGCGCTTCCCAAAGTATCTCTTCTTATCTCAACTGGTATCAACAAAAACCGGGCAAAGCGCCTAAGTTGTTGATCTACGCAGCGTCAATGCTCCAAAGCGGTGTTCCAAGCAGATTCTCCGGCAGTGGCTCAGGGACGGACTTTACGCTTACGATATCTAGCCTTCAGCCGGAAGACTTTGCAACATATTACTGCCAGCAGAATCGAGGCTTTCCACTTACTTTTGGGCAGGGTACGAAAGTGGAAATAAAAggtaccggaggttccggtggttccg

RSPO3 ΔTSP/BR HS20 (A): 1188bp

cctacgacgtgcccgactacgccaccggtaacctgCAAAACGCCTCCCGGGGAAGGCGCCAGCGAAGAATGCATCCTAACGTTAGTCAAGGCTGCCAAGGAGGCTGTGCAACATGCTCAGATTACAATGGATGTTTGTCATGTAAGCCCAGACTATTTTTTGCTCTGGAAAGAATTGGCATGAAGCAGATTGGAGTATGTCTCTCTTCATGTCCAAGTGGATATTATGGAACTCGATATCCAGATATAAATAAGTGTACAAAATGCAAAGCTGACTGTGATACCTGTTTCAACAAAAATTTCTGCACAAAATGTAAAAGTGGATTTTACTTACACCTTGGAAAGTGCCTTGACAATTGCCCAGAAGGGTTGGAAGCCAACAACCATACTATGGAGTGTGTCAGTATTGTGAGTACCGGAGGTTCAGGGGGAAGCGGAGGTTCCGGTATGGAGGTCCAACTTCTCGAAAGCGGTGGTGGTCTTGTCCAACCAGGAGGGTCACTCCGCTTGTCCTGCGCAGCTTCTGGTTTCACTTTTTCAAGCTATGCGATGAGCTGGGTGCGCCAGGCCCCTGGGAAAGGGTTGGAATGGGTCAGCACGATACAAAAACAAGGGCTTCCAACTCAATATGCAGACAGCGTGAAAGGACGGTTTACAATTTCACGAGACAATAGCAAAAATACGTTGTATTTGCAAATGAATTCACTGCGAGCGGAAGACACAGCAGTCTATTATTGTGCAGCTGCGGCTGCCGCCGCAGCAGCTTGGGGTCAAGGGACGCTCGTTACCGTTAGCAGTGGTGGAGGAGGGTCTGGGGGCGGCGGTAGCGGGGGAGGCGGAAGTGACATCCAGATGACACAAAGTCCTTCATCCCTGAGCGCAAGTGTTGGGGATCGCGTTACCATTACCTGCCGCGCTTCCCAAAGTATCTCTTCTTATCTCAACTGGTATCAACAAAAACCGGGCAAAGCGCCTAAGTTGTTGATCTACGCAGCGTCAATGCTCCAAAGCGGTGTTCCAAGCAGATTCTCCGGCAGTGGCTCAGGGACGGACTTTACGCTTACGATATCTAGCCTTCAGCCGGAAGACTTTGCAACATATTACTGCCAGCAGAATCGAGGCTTTCCACTTACTTTTGGGCAGGGTACGAAAGTGGAAATAAAAggtaccggaggttccggtggttccg

RSPO3 ΔTSP/BR HS20 (R67A/Q72A): 1188bp

cctacgacgtgcccgactacgccaccggtaacctgCAAAACGCCTCCCGGGGAAGGCGCCAGCGAAGAATGCATCCTAACGTTAGTCAAGGCTGCCAAGGAGGCTGTGCAACATGCTCAGATTACAATGGATGTTTGTCATGTAAGCCCAGACTATTTTTTGCTCTGGAAGCCATTGGCATGAAGGCCATTGGAGTATGTCTCTCTTCATGTCCAAGTGGATATTATGGAACTCGATATCCAGATATAAATAAGTGTACAAAATGCAAAGCTGACTGTGATACCTGTTTCAACAAAAATTTCTGCACAAAATGTAAAAGTGGATTTTACTTACACCTTGGAAAGTGCCTTGACAATTGCCCAGAAGGGTTGGAAGCCAACAACCATACTATGGAGTGTGTCAGTATTGTGAGTACCGGAGGTTCAGGGGGAAGCGGAGGTTCCGGTATGGAGGTCCAACTTCTCGAAAGCGGTGGTGGTCTTGTCCAACCAGGAGGGTCACTCCGCTTGTCCTGCGCAGCTTCTGGTTTCACTTTTTCAAGCTATGCGATGAGCTGGGTGCGCCAGGCCCCTGGGAAAGGGTTGGAATGGGTCAGCACGATACAAAAACAAGGGCTTCCAACTCAATATGCAGACAGCGTGAAAGGACGGTTTACAATTTCACGAGACAATAGCAAAAATACGTTGTATTTGCAAATGAATTCACTGCGAGCGGAAGACACAGCAGTCTATTATTGTGCAAAGAATAGGGCCAAGTTTGATTACTGGGGTCAAGGGACGCTCGTTACCGTTAGCAGTGGTGGAGGAGGGTCTGGGGGCGGCGGTAGCGGGGGAGGCGGAAGTGACATCCAGATGACACAAAGTCCTTCATCCCTGAGCGCAAGTGTTGGGGATCGCGTTACCATTACCTGCCGCGCTTCCCAAAGTATCTCTTCTTATCTCAACTGGTATCAACAAAAACCGGGCAAAGCGCCTAAGTTGTTGATCTACGCAGCGTCAATGCTCCAAAGCGGTGTTCCAAGCAGATTCTCCGGCAGTGGCTCAGGGACGGACTTTACGCTTACGATATCTAGCCTTCAGCCGGAAGACTTTGCAACATATTACTGCCAGCAGAATCGAGGCTTTCCACTTACTTTTGGGCAGGGTACGAAAGTGGAAATAAAAggtaccggaggttccggtggttccg

RSPO3 ΔTSP/BR HS20 (F106E/F110E): 1188bp

cctacgacgtgcccgactacgccaccggtaacctgCAAAACGCCTCCCGGGGAAGGCGCCAGCGAAGAATGCATCCTAACGTTAGTCAAGGCTGCCAAGGAGGCTGTGCAACATGCTCAGATTACAATGGATGTTTGTCATGTAAGCCCAGACTATTTTTTGCTCTGGAAGCCATTGGCATGAAGGCCATTGGAGTATGTCTCTCTTCATGTCCAAGTGGATATTATGGAACTCGATATCCAGATATAAATAAGTGTACAAAATGCAAAGCTGACTGTGATACCTGTGAGAACAAAAATGAGTGCACAAAATGTAAAAGTGGATTTTACTTACACCTTGGAAAGTGCCTTGACAATTGCCCAGAAGGGTTGGAAGCCAACAACCATACTATGGAGTGTGTCAGTATTGTGAGTACCGGAGGTTCAGGGGGAAGCGGAGGTTCCGGTATGGAGGTCCAACTTCTCGAAAGCGGTGGTGGTCTTGTCCAACCAGGAGGGTCACTCCGCTTGTCCTGCGCAGCTTCTGGTTTCACTTTTTCAAGCTATGCGATGAGCTGGGTGCGCCAGGCCCCTGGGAAAGGGTTGGAATGGGTCAGCACGATACAAAAACAAGGGCTTCCAACTCAATATGCAGACAGCGTGAAAGGACGGTTTACAATTTCACGAGACAATAGCAAAAATACGTTGTATTTGCAAATGAATTCACTGCGAGCGGAAGACACAGCAGTCTATTATTGTGCAAAGAATAGGGCCAAGTTTGATTACTGGGGTCAAGGGACGCTCGTTACCGTTAGCAGTGGTGGAGGAGGGTCTGGGGGCGGCGGTAGCGGGGGAGGCGGAAGTGACATCCAGATGACACAAAGTCCTTCATCCCTGAGCGCAAGTGTTGGGGATCGCGTTACCATTACCTGCCGCGCTTCCCAAAGTATCTCTTCTTATCTCAACTGGTATCAACAAAAACCGGGCAAAGCGCCTAAGTTGTTGATCTACGCAGCGTCAATGCTCCAAAGCGGTGTTCCAAGCAGATTCTCCGGCAGTGGCTCAGGGACGGACTTTACGCTTACGATATCTAGCCTTCAGCCGGAAGACTTTGCAACATATTACTGCCAGCAGAATCGAGGCTTTCCACTTACTTTTGGGCAGGGTACGAAAGTGGAAATAAAAggtaccggaggttccggtggttccg
