# Supplementary material for: R-spondins engage heparan sulfate proteoglycans to potentiate WNT signaling
Source: eLife. 2020 May 20;9:e54469. doi: 10.7554/eLife.54469 (PMC7239654; doi:10.7554/eLife.54469)
Supplement: Supplementary file 6. [file elife-54469-supp6.docx]

| Reagent type (species) or resource | Designation | Source or reference | Identifiers | Additional Information |
| --- | --- | --- | --- | --- |
| cell line (H. Sapiens) | WT-7TGP | Lebensohn et al., 2016 |  |  |
| cell line (H. Sapiens) | LGR4/5/6^KO^ | Lebensohn and Rohatgi, 2018 |  |  |
| cell line (H. Sapiens) | LGR4/5/6^KO^;PIGL^KO^ | Lebensohn and Rohatgi, 2018 |  |  |
| cell line (H. Sapiens) | LGR4/5/6^KO^;SDC1/2/3/4^KO^ | Lebensohn and Rohatgi, 2018 |  |  |
| cell line (H. Sapiens) | LGR4/5/6^KO^;EXTL3^KO^ | Lebensohn and Rohatgi, 2018 |  |  |
| cell line (H. Sapiens) | LGR4/5/6^KO^;GPC3^KO^ | This paper |  | Cell line maintained in Rohatgi lab and Lebensohn lab |
| cell line (H. Sapiens) | LGR4/5/6^KO^;GPC4^KO^ | This paper |  | Cell line maintained in Rohatgi lab and Lebensohn lab |
| cell line (H. Sapiens) | HEK-293T | Lebensohn and Rohatgi, 2018 |  |  |
| recombinant DNA reagent | pHLsec-HA-hRSPO3-Tev-Fc-Avi-1D4 | Lebensohn and Rohatgi, 2018 |  |  |
| recombinant DNA reagent | pHLsec-HA-hRSPO3ΔTSP/BR-Tev-Fc-Avi-1D4 | Lebensohn and Rohatgi, 2018 |  |  |
| recombinant DNA reagent | pHLsec-HA-hRSPO3TSP/BR(K/R→E)-Tev-Fc-Avi-1D4 | This paper |  | DNA sequence shown in Supplementary file 1 |
| recombinant DNA reagent | pHLsec-HA-hRSPO3ΔTSP/BRHS20-Avi-1D4 | This paper |  | DNA sequence shown in Supplementary file 1 |
| recombinant DNA reagent | pHLsec-HA-hRSPO3ΔTSP/BRHS20(GS)-Avi-1D4 | This paper |  | DNA sequence shown in Supplementary file 1 |
| recombinant DNA reagent | pHLsec-HA-hRSPO3ΔTSP/BRHS20(A)-Avi-1D4 | This paper |  | DNA sequence shown in Supplementary file 1 |
| recombinant DNA reagent | pHLsec-HA-hRSPO3ΔTSP/BRHS20(R67A/Q72A)-Avi-1D4 | This paper |  | DNA sequence shown in Supplementary file 1 |
| recombinant DNA reagent | pHLsec-HA-hRSPO3ΔTSP/BRHS20(F106E/F110E)-Avi-1D4 | This paper |  | DNA sequence shown in Supplementary file 1 |
| recombinant DNA reagent | RNF43-2xFlag-2xHA | Koo et al., 2012 |  |  |
| recombinant DNA reagent | SNAP-tagged mouse FZD5 (Ala27-Val559) | Koo et al., 2012 |  |  |
| recombinant DNA reagent | pVRC8400-GPC3ΔHS-hFc (pMH137) | Feng et al., 2013 |  |  |
| recombinant DNA reagent | pVRC8400-GPC4ΔHS-hFc (pMH373) | This paper |  | Constructed as described in Materials and methods |
| recombinant DNA reagent | pX458-mCherry | This paper |  | mCherry version of pSpCas9(BB)-2A-GFP (pX458), constructed as described in Materials and methods |
| antibody | Rho 1D4 purified monoclonal antibody | University of British Columbia | https://uilo.ubc.ca/rho-1d4-antibody |  |
| antibody | mouse anti-Flag M2 | MilliporeSigma | Cat. # F3165 |  |
| antibody | mouse anti-actin monoclonal (Clone C4) | MP Biomedicals | Cat. # 08691002 |  |
| antibody | polyclonal rabbit anti-human lysozyme | Dako, Agilent | Cat. # A0099 |  |
| antibody | rabbit anti-Ki67 | Abcam | Cat. # ab15580 |  |
| antibody | chicken anti-rabbit Alexa Fluor 488 | Thermo Fisher Scientific | Cat. # A21441 |  |
| antibody | goat anti-mouse Alexa Fluor 680 | Thermo Fisher Scientific | Cat. # A21057 |  |
| antibody | goat anti-mouse IRDye800 | Rockland Immunochemicals | Cat. # 610-132-121 |  |
| antibody | goat anti-human Ig kappa chain HRP conjugate | MilliporeSigma | Cat. # AP502P |  |
| peptide, recombinant protein | RSPO3 (WT) | Lebensohn and Rohatgi, 2018 |  |  |
| peptide, recombinant protein | RSPO3 TSP/BR (K/R→E) | This paper |  | Protein was made from plasmid pHLsec-HA-hRSPO3TSP/BR(K/R→E)-Tev-Fc-Avi-1D4; schematic is show in Figure 1B |
| peptide, recombinant protein | RSPO3 ΔTSP/BR | Lebensohn and Rohatgi, 2018 |  |  |
| peptide, recombinant protein | RSPO3 ΔTSP/BR HS20 | This paper |  | Protein was made from plasmid pHLsec-HA-hRSPO3ΔTSP/BRHS20-Avi-1D4; schematic is show in Figure 2A |
| peptide, recombinant protein | RSPO3 ΔTSP/BR HS20 (GS) | This paper |  | Protein was made from plasmid pHLsec-HA-hRSPO3ΔTSP/BRHS20(GS)-Avi-1D4; schematic is show in Figure 2A |
| peptide, recombinant protein | RSPO3 ΔTSP/BR HS20 (A) | This paper |  | Protein was made from plasmid pHLsec-HA-hRSPO3ΔTSP/BRHS20(A)-Avi-1D4; schematic is show in Figure 2A |
| peptide, recombinant protein | RSPO3 ΔTSP/BR HS20 (R67A/Q72A) | This paper |  | Protein was made from plasmid pHLsec-HA-hRSPO3ΔTSP/BRHS20(R67A/Q72A)-Avi-1D4; schematic is show in Figure 2A |
| peptide, recombinant protein | RSPO3 ΔTSP/BR HS20 (F106E/F110E) | This paper |  | Protein was made from plasmid pHLsec-HA-hRSPO3ΔTSP/BRHS20(F106E/F110E)-Avi-1D4; schematic is show in Figure 2A |
| peptide, recombinant protein | GPC3ΔHS | Feng et al., 2013 |  |  |
| peptide, recombinant protein | GPC4ΔHS | This paper |  | Protein was made as described in Materials and methods |
| peptide, recombinant protein | CD276-hFc | This paper |  |  |
| peptide, recombinant protein | Recombinant human glypican 3 protein (GPC3) | R&D Systems | Cat. # 2119-GP |  |
| peptide, recombinant protein | Recombinant human glypican 4 Fc chimera protein (GPC4) | R&D Systems | Cat. # 9195-GP |  |
| peptide, recombinant protein | Recombinant human R-Spondin 1 protein (RSPO1) | R&D Systems | Cat. # 4645-RS |  |
| peptide, recombinant protein | Recombinant human R-Spondin 3 protein (RSPO3) | R&D Systems | Cat. # 3500-RS |  |
| peptide, recombinant protein | WNT3A conditioned medium (CM) | Lebensohn et al., 2016 |  |  |
| peptide, recombinant protein | recombinant murine EGF | Peprotech | Cat. # 315-09 |  |
| peptide, recombinant protein | Noggin CM | Sato et al., 2009 |  |  |
| other | NEBuilder HiFi DNA Assembly Master Mix | New England Biolabs | Cat. # E2621L |  |
| other | phalloidin-TRITC | MilliporeSigma | Cat. # P1951 |  |
| other | DAPI | MilliporeSigma | Cat. # D9542 |  |
| other | Dulbecco’s Modified Eagle’s Medium (DMEM)/high glucose without L-glutamine, sodium pyruvate | HyClone, GE Healthcare Life Sciences | Cat. # SH30081.FS |  |
| other | Iscove's Modified Dulbecco's Medium (IMDM) modified medium with L-glutamine, HEPES | HyClone, GE Healthcare Life Sciences | Cat. # SH30228.FS |  |
| other | Advanced DMEM/F12 | Gibco, Thermo Fisher Scientific | Cat. # 12634010 |  |
| other | CD 293 medium (1X) | Gibco, Thermo Fisher Scientific | Cat. # 11913019 |  |
| other | Fetal bovine serum | MilliporeSigma | Cat. # S11150 |  |
| other | Sodium pyruvate (100 mM) | Gibco, Thermo Fisher Scientific | Cat. # 11360070 |  |
| other | MEM non-essential amino acids (100X) | Gibco, Thermo Fisher Scientific | Cat. # 11140050 |  |
| other | L-glutamine solution (stabilized) | Gemini Bio-products | Cat. # 400-106 |  |
| other | Penicillin: streptomycin solution | Gemini Bio-products | Cat. # 400-109 |  |
| other | HEPES (1M) | Gibco, Thermo Fisher Scientific | Cat. # 15630080 |  |
| other | GlutaMAX Supplement | Gibco, Thermo Fisher Scientific | Cat. # 35050061 |  |
| other | N-2 Supplement (100X) | Gibco, Thermo Fisher Scientific | Cat. # 17502001 |  |
| other | B-27™ Supplement (50X), serum free | Gibco, Thermo Fisher Scientific | Cat. # 17504044 |  |
| other | Trypsin-EDTA (0.05%), phenol red | Gibco, Thermo Fisher Scientific | Cat. # 25300054 |  |
| other | Polyethylenimine, linear, MW 25,000, Transfection Grade (PEI 25K) | Polysciences, Inc. | Cat. # 23966-1 |  |
| other | Valproic acid sodium salt | MilliporeSigma | Cat. # P4543 |  |
| other | Rho 1D4 resin | Lebensohn and Rohatgi, 2018 |  |  |
| other | 1D4 peptide ((NH3)-T-E-T-S-Q-V-A-P-A-(COOH)) | Lebensohn and Rohatgi, 2018 |  |  |
| other | EZ-Link Sulfo-NHS-SS-Biotin | Thermo Fisher Scientific | Cat. # 21331 |  |
| other | Streptavidin agarose beads | Pierce, Thermo Fisher Scientific | Cat. # 20349 |  |
| other | 3,3′,5,5′-tetramethylbenzidine detection reagent | Kirkegaard & Perry Laboratories | Cat. # 95059-156 |  |
| other | N-acetylcysteine | MilliporeSigma | Cat. # 106425 |  |
| other | Rapid-Flow sterile disposable filter units with aPES membrane, 0.45μm | Nalgene, Thermo Fisher Scientific | Cat. # 166-0045 |  |
| other | Immobilon-FL PVDF membranes | MilliporeSigma | Cat. # IPFL00005 |  |
| other | Odyssey blocking buffer | Li-Cor | Cat. # 927-40000 |  |
| other | Protein A Hi-Trap column | GE Healthcare | Cat. # 29048576 |  |
| other | Corning CellBIND microplates (96-well) | Corning | Cat. # 3300 |  |
| other | Corning CellBIND multiple well plates (6-well) | Corning | Cat. # 3335 |  |
| other | Corning CellBIND culture flasks | Corning | Cat. # 3292 |  |
| other | μ-slide 8 well | Ibidi | Cat. # 80826 |  |
| other | Ibidi mounting medium | Ibidi | Cat. # 50001 |  |
